# The localization of non-backtracking centrality in networks and its physical consequences

**DOI:** 10.1038/s41598-020-78582-x

**Published:** 2020-12-10

**Authors:** Romualdo Pastor-Satorras, Claudio Castellano

**Affiliations:** 1 Departament de Física, Universitat Politècnica de Catalunya, Campus Nord B4, 08034 Barcelona, Spain; 2 Istituto dei Sistemi Complessi (ISC-CNR), Via dei Taurini 19, 00185 Roma, Italy

**Keywords:** Complex networks, Nonlinear phenomena, Phase transitions and critical phenomena

## Abstract

The spectrum of the non-backtracking matrix plays a crucial role in determining various structural and dynamical properties of networked systems, ranging from the threshold in bond percolation and non-recurrent epidemic processes, to community structure, to node importance. Here we calculate the largest eigenvalue of the non-backtracking matrix and the associated non-backtracking centrality for uncorrelated random networks, finding expressions in excellent agreement with numerical results. We show however that the same formulas do not work well for many real-world networks. We identify the mechanism responsible for this violation in the localization of the non-backtracking centrality on network subgraphs whose formation is highly unlikely in uncorrelated networks, but rather common in real-world structures. Exploiting this knowledge we present an heuristic generalized formula for the largest eigenvalue, which is remarkably accurate for all networks of a large empirical dataset. We show that this newly uncovered localization phenomenon allows to understand the failure of the message-passing prediction for the percolation threshold in many real-world structures.

## Introduction

The non-backtracking (NB) operator is a binary matricial representation of the topology of a network, whose elements represent the presence of *non-backtracking* paths between pairs of different nodes, traversing a third intermediate one^1,2^. By means of a message-passing approach^3^, the NB matrix finds a natural use in the representation of dynamical processes on networks, such as percolation^4,5^ and non-recurrent epidemics^6^, where a spreading process cannot affect twice a given node, and therefore backtracking propagation paths are inhibited^7,8^. Within this approach, the bond percolation threshold and the epidemic threshold in the SIR model^6^ are found to be inversely proportional to the largest eigenvalue (LEV) of the NB matrix, $\mu _M$. The spectrum of the non-backtracking matrix is relevant also for other problems in network science, such as community structure^9^ and node importance^2,10–12^.

The principal eigenvector (PEV) associated to the LEV of the NB matrix has been recently used to build a new measure of node importance or centrality^13^. A classical measure of node centrality is given by eigenvector centrality, based on the idea that a node is central if it is connected to other central nodes. In this perspective, eigenvector centrality of node *i* is defined as the *i*th component of the principal eigenvector of the adjacency matrix^14^. Eigenvector centrality has the drawback of being strongly affected by the presence of large hubs, which exhibit an exceedingly large component of the adjacency matrix PEV because of a peculiar self-reinforcing bootstrap effect. The hub is highly central since it has a large number of mildly central neighbors; the neighbors are in their turn central just because of their vicinity with the highly central hub^2,15^. In terms of the adjacency matrix this self-reinforcement is revealed by the localization of the PEV on a star graph composed by the largest hub and its immediate neighbors. To correct for this feature, in Ref.^2^ it was proposed to build a centrality measure using the NB matrix, in such a way as to avoid backtracking paths that could artificially inflate a hub’s centrality. In this way, an alternative non-backtracking centrality (NBC) of nodes was defined, in which the effect of hubs is strongly suppressed.

Consider an unweighted undirected complex network with *N* nodes and *E* edges. The non-backtracking (NB) matrix ${\mathbf {B}}$ is a representation of the network topology in terms of a $2E \times 2E$ non-symmetric matrix in which rows and columns represent virtual directed edges $j \rightarrow i$ pointing from node *j* to node *i*, taking the value

1 $$\begin{aligned} B_{j \rightarrow i, m \rightarrow \ell } = \delta _{j \ell } ( 1- \delta _{i m}), \end{aligned}$$

where $\delta _{i j}$ represents the Kronecker symbol. Each NB matrix element represents a possible walk in the network composed by a pair of directed edges, one pointing from node *m* to node $\ell$, and the other from node *j* to node *i*. The element is nonzero when the edges share the central node ($j = \ell$), and when the walk does not return to the first node ($m \ne i$).

The principal eigenvector $v_{j \rightarrow i}$ of the NB matrix, associated to the largest eigenvalue (LEV) $\mu _M$, is given by the relation

2 $$\begin{aligned} \mu _M v_{j \rightarrow i} = \sum _{m \rightarrow l} B_{j \rightarrow i, m \rightarrow l} v_{m \rightarrow l}. \end{aligned}$$

Since ${\mathbf {B}}$ is a non-negative matrix, the Perron–Frobenius theorem^16^ guarantees that $\mu _M$ and all components $v_{j \rightarrow i}$ are positive, provided that the matrix is irreducible.

The element $v_{j \rightarrow i}$ expresses the centrality of node *j*, disregarding the possible contribution of node *i*. The non-backtracking centrality $x_i$ of node *i* is defined as^2^

3 $$\begin{aligned} x_i = \sum _j A_{ij} v_{j \rightarrow i}, \end{aligned}$$

where $A_{ij}$ is the network adjacency matrix. If the PEV of the NB matrix is normalized as $\sum _{j \rightarrow i} v_{j \rightarrow i} = \sum _{j, i} A_{ji} v_{j \rightarrow i} = 1$, which is valid if ${\mathbf {B}}$ is irreducible, then the natural normalization $\sum _i x_i = 1$ emerges.

## Results

### Theory for uncorrelated random networks

The NBC can be practically calculated by using the Ihara–Bass determinant formula^2,17^, which shows that the NBC values $x_i$ correspond to the first *N* elements of the PEV of the $2N \times 2N$ matrix

4 $$\begin{aligned} {\mathbf {M}} = \left( \begin{array}{cc} {\mathbf {A}} &{} {\mathbf {I}} - {\mathbf {D}}\\ {\mathbf {I}} &{} {\mathbf {0}} \end{array} \right) , \end{aligned}$$

where ${\mathbf {A}}$ is the adjacency matrix, ${\mathbf {I}}$ is the identity matrix, and ${\mathbf {D}}$ is a diagonal matrix of elements $D_{ij} = \delta _{ij} k_i$. Using the Ihara–Bass formalism^18^ (see Method “Theory for uncorrelated networks” section) one can express, in full generality, the leading eigenvalue $\mu _M$ in terms of the NBC as

5 $$\begin{aligned} \mu _M = \frac{\sum _i k_i x_i}{\sum _i x_i} -1. \end{aligned}$$

Following Ref.^2^ (see Method “Theory for uncorrelated networks” section), it is possible to argue that, for uncorrelated random networks, i.e., networks with a given degree sequence but completely random in all other respects^13^, the dependence of the components of the NB matrix PEV is

6 $$\begin{aligned} v_{j \rightarrow i} \sim k_j-1 . \end{aligned}$$

Introducing this relation into the definition of the NBC, Eq. (3), and applying the normalization $\sum _i x_i = 1$, we obtain

7 $$\begin{aligned} x_i^{\mathrm{un}} = \frac{\sum _j A_{ij} (k_j - 1)}{\sum _j k_j(k_j - 1)}, \end{aligned}$$

that, inserted into Eq. (5), leads to

8 $$\begin{aligned} \mu _M^{\mathrm{un}} = \frac{\sum _{ij} (k_i - 1) A_{ij} (k_j -1)}{\sum _j k_j(k_j - 1)}. \end{aligned}$$

These expressions constitute an improvement over previous results^2,9,18^, namely

9 $$\begin{aligned} x_i^{\mathrm{an}} = \frac{k_i}{\left\langle {k} \right\rangle N}, \quad {\mathrm {and}} \quad \mu _M^{\mathrm{an}} = \frac{\left\langle {k^2} \right\rangle }{\left\langle {k} \right\rangle } - 1, \end{aligned}$$

($\left\langle {k^n} \right\rangle$ is the *n*th moment of the degree distribution), which can be recovered from Eqs. (7) and (8) by replacing the network adjacency matrix with its annealed approximated value ${\bar{A}}_{ij} = k_i k_j/(\left\langle {k} \right\rangle N)$^19,20^.

### Test on synthetic networks

**Figure 1:**
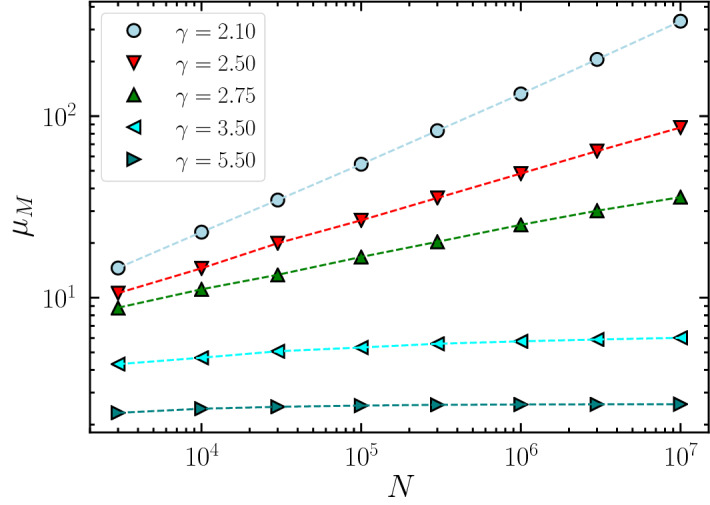
$\mu _M$ for uncorrelated networks. Scaling of the LEV of the NB matrix, $\mu _M$, as a function of network size *N* in power law UCM networks with different degree exponent $\gamma$. Dashed lines correspond to the theoretical prediction Eq. (8). Simulations results correspond to the average over 25 different network realizations. Error bars are smaller than symbols size.

**Figure 2:**
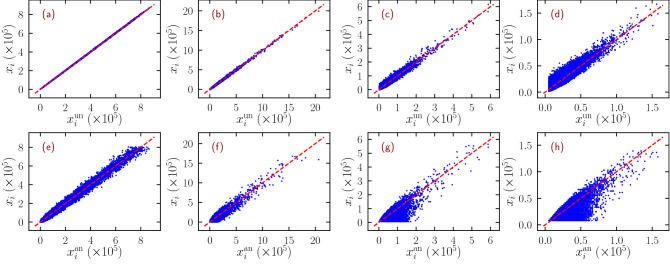
NBC for uncorrelated networks. Scatter plot of the numerical NBC $x_i$ in power-law UCM networks of size $N=10^6$ with different degree exponent $\gamma$, as a function of the theoretical predictions $x_i^{\mathrm{un}}$ in Eq. (7) (top row) and $x_i^{\mathrm{an}}$ in Eq. (9) (bottom row). The dashed lines represent the curve $y = x$. Degree exponents considered are $\gamma = 2.10$ (**a**) and (**e**); $\gamma = 2.75$ (**b**) and (**f**); $\gamma = 3.50$ (**c**) and (**g**); $\gamma = 4.50$ (**d**) and (**h**).

We now check the predictions developed above with the LEV $\mu _M$ and the NBC $x_i$ determined numerically by applying the power iteration method^21^ to the Ihara–Bass matrix ${\mathbf {M}}$ for random uncorrelated networks with a power-law degree distribution $P(k) \sim k^{-\gamma }$, generated using the uncorrelated configuration model (UCM)^22^. In Fig. 1 we present, as a function of the network size *N*, a comparison between the NB LEV, $\mu _M$, evaluated numerically and our theoretical prediction Eq. (8). The match between theory and simulation is excellent. However, also Eq. (9) gives very accurate results, differing in average by less than $0.5\%$ from the theoretical result Eq. (8). A much more noticeable improvement is observed instead for the NB centrality $x_i$, for which annealed network approximation does not provide accurate predictions (see Fig. 2, bottom row). In Fig. 2 (top row) we show the dependence of the NBC $x_i$ on the structure of the adjacency matrix, as given by Eq. (7), namely $x_i \sim \sum _j A_{ij} (k_j -1)$. The analytical expression is extremely accurate for values of $\gamma < 3$. For $\gamma > 3$, although some scattering can be observed with respect to the expected value, the prediction is still good, much more accurate than the annealed network approximation. More evidence about the superior accuracy of our approach is found considering the inverse participation ratio $Y_4(N)$ as a function of network size (see Method “Localization of the non-backtracking centrality”).

### Non-backtracking principal eigenvalue of characteristic subgraphs

The non-backtracking centrality was introduced with the goal of overcoming the flaws of eigenvector centrality, due to the localization of the adjacency matrix principal eigenvector on star graphs surrounding hubs of large degree, that artificially inflate their own eigenvector centrality^2^. For the NBC the addition of a large hub to an otherwise homogeneous network has a limited impact. Indeed, the addition of a dangling hub of degree *K*, connected to $K-1$ leaves of degree 1 and to a generic network by a single edge, does not alter at all the value of $\mu _M$^2,9^ (see Method “Largest non-backtracking eigenvalue of characteristic subgraphs” section). In the case of a hub integrated into the network, connected to *K* other random nodes in the graph, Ref.^2^ argued, from the perspective of the annealed network approximation, that its effect is irrelevant in the thermodynamic limit. A more elaborate analysis (see Method “Largest non-backtracking eigenvalue of characteristic subgraphs” section) shows that this is true unless $K \gg (N/\left\langle {k} \right\rangle )^{1/2}$. Only in this case an integrated hub has an effect and leads to a PEV significantly larger than the PEV of the original network and scaling as $[\left\langle {k} \right\rangle K(K-1)/N]^{1/3}$.

However, it is possible that other types of subgraphs play for the NB centrality the same role that star graphs play for eigenvector centrality: They can have, alone, large values of $\mu _M$, so that, if present within an otherwise random network, they determine $\mu _M$ of the whole structure, with the overall NBC localized on them. We now show that these subgraphs actually exist and can have dramatic effects.

As noticed in Ref.^2^, the simplest example is a clique of size $K_c$, which is associated to $\mu _M^{\mathrm{clique}}=K_c-2$. If $K_c$ is large enough, $\mu _M^{\mathrm{clique}}$ can dominate over $\mu _M^{\mathrm{un}}$. But also a homogeneous (Poisson) subgraph of average degree $\left\langle {k} \right\rangle$, for which $\mu _M=\left\langle {k} \right\rangle$^2,9^, can become the substrate of a localized NB PEV if $\left\langle {k} \right\rangle$ is sufficiently large.

Apart from these simple examples, a less trivial one is the case of *overlapping hubs*, i.e., a set of *n* hubs of degree *K*, connected to the same *K* leaves of degree *n*, see Supplementary Fig. SF1. The intrinsic LEV associated to such a structure is (see Method “Largest non-backtracking eigenvalue of characteristic subgraphs section”)

10 $$\begin{aligned} \mu _M^{{\mathrm {oh}}} = \sqrt{(n-1)(K-1)}. \end{aligned}$$

This last case is particularly important, since $\mu _M^{{\mathrm {oh}}}$ can become very large due to a few overlapping hubs of very large degree *K*, or due to a large number of hubs with moderate overlap *K*.

### Localization in real-world networks

**Figure 3:**
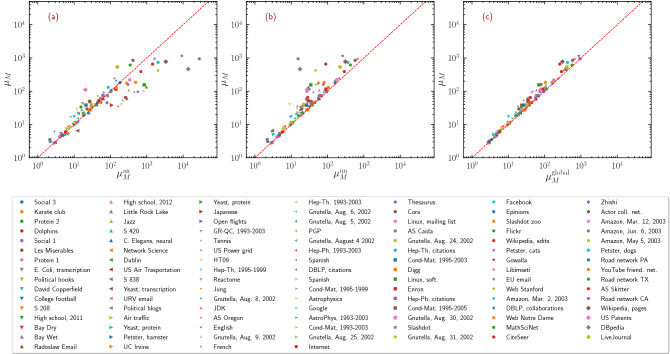
Test of theoretical approaches for real-world networks. LEV of the NB matrix, $\mu _M$, as a function of the theoretical predictions $\mu _M^{\mathrm{un}}$ [Eq. (8a)] $\mu _M^{\mathrm{an}}$ [Eq. (9b)], and $\mu _M^{\mathrm {global}}$ [Eq. (11c)], for the set of 109 real-world networks described in Supplementary Table ST1.

In Fig. 3a,b we compare the theoretical predictions derived for uncorrelated and annealed networks with the values of $\mu _M$ computed numerically for a set of 109 real-world networks of diverse origin (see Supplementary Table ST1 for details). In opposite ways, both predictions, $\mu _M^{\mathrm{an}}$ and $\mu _M^{\mathrm{un}}$, fail to provide an accurate approximation of empirical results for many networks. In the most noticeable cases, the networks Zhishi and DBpedia, the uncorrelated prediction Eq. (8) largely underestimates the value of $\mu _M$, while the annealed network prediction Eq. (9) largely overestimates it.

To shed light on the origin of these discrepancies, in Supplementary Fig. SF2 we compare the empirical NBC, $x_i$, with the theoretical prediction $x_i^{\mathrm{un}}$ for four real-world networks in which the predictions largely fail. We observe that, in all networks, a few nodes assume an exceedingly large value of $x_i$, i.e., the NBC is localized on a very small subset of nodes, which includes the largest hubs.

It is clear that, in order to obtain an accurate prediction of $\mu _M$ in real-world networks, it is necessary to take into account the possible localization of the NB centrality on subgraphs which, despite being relatively small, may determine $\mu _M$ for the whole structure. In previous paragraphs, we have seen that two special subgraphs, a large clique/relatively dense homogeneous graph, or a set of overlapping hubs, may become the set where NBC gets localized if the associated $\mu _M$ is larger than the one for the rest of the network. It is then natural to postulate (in analogy with what happens for the adjacency matrix^23^) that the overall $\mu _M$ is well approximated by the maximum among Eq. (8) and the $\mu _M^{(s)}$ values associated to each possible network subgraph *s* (We note here that, while in the case of the adjacency matrix this result is exact due to the Rayleigh’s inequality^24^, for the NB matrix we simply proceed by analogy. As we will see later on, however, the conjecture turns out to be quite accurate). An exhaustive search among all subgraphs is computationally impractical. However, if we limit ourselves to the types of subgraphs discussed above, it is numerically easy to find reasonable estimates of their maximum LEVs. The hubs, either dangling or integrated, provide a negligible contribution, as we can check numerically. The *K*-core decomposition (see Method “*K*-core decomposition”) provides, as the core with maximum index, an approximation of the densest subgraph in the network. The value $\mu _M^{{\mathrm {core}}}$ associated to such max *K*-core, which can be either a clique or a relatively dense homogeneous graph, is a good estimate of the maximum LEV among these types of subgraphs. Concerning $\mu _M^{{\mathrm {oh}}}$, the pair of *n* and *K* values maximizing Eq. (10) can be well approximated by a heuristic greedy algorithm described in Method “Algorithm to determine optimal *n* and *K* values for overlapping hubs”.

Following this line of reasoning, we can then write an approximate expression for the NB LEV in generic networks as

11 $$\begin{aligned} \mu _M^{\mathrm {global}} = \max \left\{ \mu _M^{\mathrm{un}}, \, \mu _M^{{\mathrm {oh}}}, \, \mu _M^{{\mathrm {core}}} \right\} , \end{aligned}$$

where $\mu _M^{{\mathrm {core}}}$ is computed as the largest eigenvalue of the NB matrix defined by the subgraph spanned by the maximum *K*-core. The comparison of Eq. (11) with empirical results in real-world networks, displayed in Fig. 3c, reveals a striking accuracy in all cases and substantiates the predictive power of Eq. (11) for the LEV of the non-backtracking matrix on generic real-world networks. The spontaneous formation of large cliques or sets of overlapping hubs is exceedingly improbable in uncorrelated networks. A *K*-core structure exists only for $\gamma <3$^25^ but in that case $\mu _M^{{\mathrm {core}}} \simeq \mu _M^{\mathrm{un}}$. As a consequence, for all uncorrelated networks Eq. (11) gives back Eq. (8).

### Application to percolation

Spectral properties of the non-backtracking matrix are at the heart of the message-passing theory for bond percolation^7^: For locally tree-like networks, the percolation threshold is given by the inverse of the NB matrix LEV,

12 $$\begin{aligned} p_c = \frac{1}{\mu _M}. \end{aligned}$$

**Figure 4:**
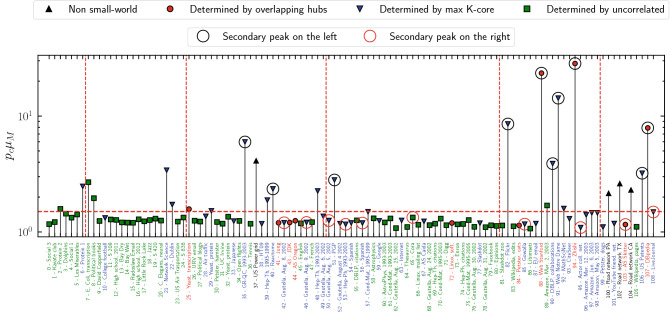
Test of message-passing prediction for bond percolation threshold in real-world networks. The bond percolation threshold $p_c$ determined numerically from the main peak of the susceptibility is divided by the message-passing prediction [Eq. (12)] and plotted for the 109 real-world networks considered. Below the horizontal dashed red line the prediction is accurate within $50\%$. Vertical dashed lines represent the size scale of the networks: from left to right $N=10^2$, $10^3$, $10^4$, $10^5$, and $10^6$. Symbols show which of the terms in Eq. (11) is maximal. Symbols are surrounded by a black (red) circle in case a secondary peak appears in the susceptibility on the left (right) of the main peak.

**Figure 5:**
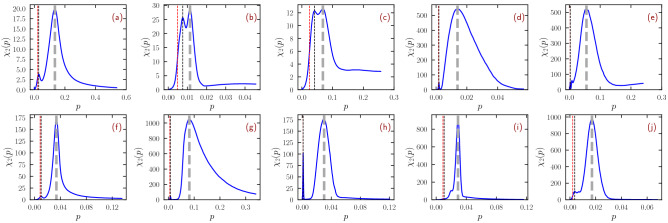
Susceptibility plots for networks exhibiting a secondary peak on the left. Numerical bond percolation susceptibility for the networks (**a**): GR-QC, 1993-2003; (**b**): Reactome; (**c**): PGP; (**d**): Flickr; (**e**): Web Stanford; (**f**): DBLP, collaborations; (**g**): Web Notre Dame; (**h**): Zhishi; (**i**): US Patents; and (**j**): DBpedia. The global maximum of the susceptibility $\chi _2(p)$, indicating the percolation threshold, is marked by a gray vertical bar. Black vertical lines indicate the position of the secondary peak. Red vertical lines signal the value of the prediction $1/\mu _M$. Notice that for three of the networks (Web Stanford, Zhishi and DBpedia) the NBC is localized on overlapping hubs, while for the others localization occurs on the max K-core.

A comparison of this prediction with results obtained numerically for our set of real-world networks is presented (A similar test was already performed in Ref.^18^.) in Fig. 4, where the percolation threshold $p_c$ is obtained as the position of the main susceptibility peak (see Method “Numerical simulations of bond percolation”). In the majority of cases $p_c$ and $1/\mu _M$ differ by less than 50%, but for the remaining networks the discrepancy is larger, in some cases by more than one order of magnitude. These failures of prediction (12) can be understood by applying the knowledge acquired in the previous Sections. Most (and the largest) of the violations occur when the NBC is localized on small subgraphs, either overlapping hubs or the max K-core, which determine the overall value of $\mu _M$. In these cases the system actually undergoes what can be seen as a double percolation transition^26^, reflected, in Fig. 5, by the presence of two distinct peaks of the susceptibility $\chi _2(p)$ (see also Ref.^27^ for the effect of mesoscopic structures on percolation). In the networks considered in this figure, the message-passing value $p=1/\mu _M$ signals the buildup of the connected subgraph of relatively small size where NBC is localized, originating the first susceptibility peak. The second and largest peak occurs for much larger values of *p* and signals the formation of a percolating cluster encompassing a larger fraction of the nodes. Two (or even multiple) peaks are present also in other networks. The message-passing theory accurately predicts only the leftmost of these peaks (see Fig. 5), while it does not give any information about the position of other peaks and the associated transition.

Some other networks exhibit quite large discrepancies between $p_c$ and $1/\mu _M$ but in the absence of a secondary peak. Our theory does not provide an explanation for these cases. However, it must be remarked that this phenomenology occurs for small networks, for which the very concept of localization on a subgraph is not well defined. Moreover, in these cases the peak of the susceptibility is wide and it may hide the presence of another peak (see Supplementary Fig. SF3).

Finally, an ample discrepancy between $p_c$ and $1/\mu _M$ is observed also for a few networks (Road network TX, Road 512 network CA, Road network PA and US Power grid) having very large values of the average shortest path length $\left\langle {\ell } \right\rangle$ and thus not possessing the small-world property. This is not surprising, as the almost planar nature of these topologies makes our framework inapplicable to them.

In summary, realizing that localization of the NB centrality can determine the value of $\mu _M$ for the whole structure allows us to understand the presence of a double percolation transition in several real-world networks. In these cases message-passing theory captures only the first of the transitions, corresponding to the emergence of a localized subgraph, while the occurrence of the second transition is completely missed by the theory^28,29^.

## Discussion

Our results show that the non-backtracking centrality, which was introduced to avoid the pathological self-reinforcement mechanism that plagues standard eigenvector centrality, is affected by the same problem. The NBC may also get localized on specific network subgraphs, with the same bootstrap mechanism at work: Some nodes are highly central because they are in “contact” with other central nodes and the latter are central because they are in contact with the former. The only difference is that for the adjacency matrix the relevant subgraphs are stars and self-reinforcement takes place among the hub and its direct neighbors^23^. For the NB matrix the relevant subgraphs are groups of nodes sharing many neighbors and self-reinforcement occurs at distance 2. The possibility of localization also for the NB matrix was overlooked so far, because it is exceedingly unlikely in random uncorrelated networks. However, as we show here, in real-world topologies these structures are rather common. Indeed, cliques and sets of overlapping hubs are, respectively, complete unipartite and bipartite subgraphs, which naturally arise in many networks, for structural or functional reasons.

The results presented here have a number of implications. Which of the three contributions determines $\mu _M^{\mathrm {global}}$ in Eq. (11) allows to rapidly estimate also the relevant non-backtracking centralities in the network. If $\mu _M^{\mathrm{un}}$ dominates, then the NBC are given by Eq. (7). If instead $\mu _M^{{\mathrm {oh}}}$ is largest, then non-backtracking centralities are given by Eq. (41) in the subset of overlapping hubs and are essentially zero elsewhere. Similarly, when $\mu _M^{{\mathrm {core}}}$ dominates in Eq. (11), NBC is approximately constant in the max K-core and much smaller elsewhere. Additionally, our results allow to shed light on the LEV of the adjacency matrix, $\Lambda _M$. In Ref.^23^, it was argued that $\Lambda _M$ is determined by two subgraphs that have associated a large LEV, and that correspond to the node of maximum degree $k_{\max}$ (hub), taken as an isolated star graph, and the maximum *K*-core. Thus, in the spirit of Rayleigh’s inequality^24^, it was proposed the approximation $\Lambda _M \simeq \max \{ \sqrt{k_{\max}}, \Lambda _{M}^{{\mathrm {core}}} \}$, where $\sqrt{k_{\max}}$ is the LEV of star graph of degree $k_{\max}$ and $\Lambda _{M}^{{\mathrm {core}}}$ is the LEV of the maximum *K*-core, approximated by its average degree $\left\langle {k} \right\rangle _{{\mathrm {core}}}$^23^. The subgraph composed by *n* overlapping hubs of degree *K* turns out to possess also a large LEV of the adjacency matrix, given by $\Lambda _M^{{\mathrm {oh}}} = \sqrt{nK}$. We can then propose an improved approximation, taking into account the effect of overlapping hub, of the form $\Lambda _M \simeq \max \{ \sqrt{k_{\max}}, \Lambda _{M}^{{\mathrm {core}}}, \Lambda _M^{\mathrm {oh}}\}$. In Supplementary Fig. SF4 we check this new expression, observing that it provides some improvement in the estimation of the adjacency matrix LEV, particularly for networks of large size.

The localization phenomenon of the NB matrix has also strong implications for percolation and thus for the related susceptible-infected-removed model for epidemic dynamics. Quite surprisingly, this reveals strong analogies with what happens in some regions of the phase-diagram of the paradigmatic susceptible-infected-susceptible model for epidemic dynamics (SIS)^30^. The formation (under appropriate conditions) of localized clusters below the global epidemic transition is a striking common feature of both types of dynamics, which they share despite their completely different nature. This intriguing similarity extends to the predictive power of theoretical approaches. For SIS dynamics quenched mean-field theory predicts when localized clusters of activity start to appear, but misses the formation of an overall endemic state^30^. For percolation (and SIR dynamics) message-passing theory captures the formation of localized clusters but is not predictive for what concerns the possible second transition involving a much larger fraction of the network. The quest for theoretical approaches able to understand and predict this nontrivial second transition is a challenging avenue for future research.

Another related line for future research is the exploitation of the improved understanding presented here to devise targeted immunization strategies^12^.

## Methods

### Theory for uncorrelated networks

Denoting the PEV of the matrix ${\mathbf {M}}$ as $\vec {f} = \{ \vec {x}, \vec {w} \}$, we can rewrite Eq. (4) as^18^

13 $$\begin{aligned} \sum _j A_{ij} x_j + w_i - k_i w_i&= \mu _M x_i, \end{aligned}$$

14 $$\begin{aligned} x_i&= \mu _M w_i, \end{aligned}$$

which translates into

15 $$\begin{aligned} \mu _M \sum _j A_{ij} x_j + x_i - k_i x_i = \mu _M^2 x_i . \end{aligned}$$

Summing over *i* and rearranging, we obtain

16 $$\begin{aligned} (\mu _M -1) \sum _i k_i x_i = (\mu _M^2 -1) \sum _i x_i . \end{aligned}$$

Discarding the solution $\mu _M = 1$, which is always an eigenvalue, we have

17 $$\begin{aligned} \sum _i k_i x_i = (\mu _M + 1) \sum _i x_i , \end{aligned}$$

leading to

18 $$\begin{aligned} \mu _M = \frac{\sum _i k_i x_i}{\sum _i x_i} -1, \end{aligned}$$

which allows us to compute $\mu _M$ once the NBC is known.

Following Ref.^2^, we can obtain an approximation for the NB matrix PEV (and hence for the NBC) by expanding the eigenvalue relation

19 $$\begin{aligned} \mu _M v_{k \rightarrow l} = \sum _{i \rightarrow j} B_{k \rightarrow l, i \rightarrow j} v_{i \rightarrow j}, \end{aligned}$$

that, after some transformations can be written as^2^

20 $$\begin{aligned} \mu _M v_{i \rightarrow l} = \sum _j A_{ij} (1 - \delta _{jl}) v_{j \rightarrow i} = \sum _{j \ne l} A_{ij} v_{j \rightarrow i} . \end{aligned}$$

Let us now compute the average value of $v_{i \rightarrow l}$ over all outgoing nodes *i* with a fixed degree $k_i = k$, that is

21 $$\begin{aligned} v_{\mathrm {out}}(k) = \frac{1}{k N P(k)} \sum _{\begin{array}{c} i \rightarrow l\\ k_i = k \end{array}} v_{i \rightarrow l} = \frac{1}{k N P(k)} \sum _{\begin{array}{c} i,l \\ k_i = k \end{array}} A_{il} v_{i \rightarrow l}, \end{aligned}$$

where *kNP*(*k*) represents the number of edges emanating from nodes of degree *k*. Applying Eq. (20) to the previous equation we can write

22 $$\begin{aligned} v_{\mathrm {out}}(k)&= \frac{1}{k N P(k) \mu _M} \sum _{\begin{array}{c} i,l\\ k_i = k \end{array}} \sum _{j \ne l} A_{ij} A_{il} v_{j \rightarrow i} \end{aligned}$$

23 $$\begin{aligned}&= \frac{1}{k N P(k) \mu _M} \sum _{\begin{array}{c} i,j\\ k_i = k \end{array}} A_{ij} v_{j \rightarrow i} \sum _{l \ne j} A_{il} \end{aligned}$$

24 $$\begin{aligned}&= \frac{k - 1}{k N P(k) \mu _M} \sum _{\begin{array}{c} i,j\\ k_i = k \end{array}} A_{ij} v_{j \rightarrow i}. \end{aligned}$$

Assuming now^2^ that the components $v_{j \rightarrow i}$ departing from nodes of degree $k_i = k$ have the same distribution as in the whole network (assumption valid in the limit of random uncorrelated networks), we can substitute $v_{j \rightarrow i} \simeq \left\langle {v} \right\rangle = \sum _{i \rightarrow j} v_{i \rightarrow j} / (2E)$, where *E* is the number of undirected edges in the original network. With this assumption, we can write

25 $$\begin{aligned} v_{\mathrm {out}}(k)&\simeq \frac{\left\langle {v} \right\rangle (k - 1)}{k N P(k) \mu _M} \sum _{\begin{array}{c} i,j\\ k_i = k \end{array}} A_{ij} \nonumber \\&= \frac{\left\langle {v} \right\rangle (k - 1)}{k N P(k) \mu _M} k N P(k) = \frac{\left\langle {v} \right\rangle }{\mu _M} (k-1). \end{aligned}$$

Analogously, we can compute the average of $v_{i \rightarrow l}$ over all ingoing nodes *l* with fixed degree $k_l = k$,

26 $$\begin{aligned} v_{\mathrm {in}}(k) = \frac{1}{k N P(k)} \sum _{\begin{array}{c} i \rightarrow l\\ k_l = k \end{array}} v_{i \rightarrow l} = \frac{1}{k N P(k)} \sum _{\begin{array}{c} i,l \\ k_l = k \end{array}} A_{il} v_{i \rightarrow l}. \end{aligned}$$

Applying again Eq. (20), we can write

27 $$\begin{aligned} v_{\mathrm {in}}(k)&= \frac{1}{k N P(k) \mu _M} \sum _{\begin{array}{c} i, l\\ k_l = k \end{array}} \sum _{j \ne l} A_{il} A_{ij} v_{j \rightarrow i} \nonumber \\&\simeq \frac{\left\langle {v} \right\rangle }{k N P(k) \mu _M} \sum _{\begin{array}{c} l\\ k_l = k \end{array}} \sum _{j \ne l} \sum _i A_{li}A_{ij} \nonumber \\&\simeq \frac{\left\langle {v} \right\rangle }{k N P(k) \mu _M} \sum _{\begin{array}{c} l\\ k_l = k \end{array}} \sum _{j \ne l} (A^2)_{lj}. \end{aligned}$$

The matrix element $(A^2)_{lj}$ counts the number of walks of length 2 between nodes *l* and *j*^13^, and

$$\begin{aligned} \sum _{\begin{array}{c} l\\ k_l = k \end{array}} \sum _{j \ne l} (A^2)_{lj} \end{aligned}$$

counts those walks that start at nodes of degree *k* and are non-backtracking. In a tree-like network, the number of such walks is equal to the number of next-nearest neighbors of nodes of degree *k*, that is in average $k N P(k) (\left\langle {k^2} \right\rangle - \left\langle {k} \right\rangle ) / \left\langle {k} \right\rangle$^13^. Therefore, we have

28 $$\begin{aligned} v_{\mathrm {in}}(k) \simeq \frac{\left\langle {v} \right\rangle }{\mu _M} \frac{\left\langle {k^2} \right\rangle - \left\langle {k} \right\rangle }{\left\langle {k} \right\rangle }. \end{aligned}$$

That is, in random uncorrelated networks, we have $v_{\mathrm {out}}(k) \sim k - 1$ and $v_{\mathrm {in}}(k) \sim {\mathrm {const.}}$. Extending this relation at the level of individual edges, we can approximate the normalized dependence of the components of the NB matrix PEV as

29 $$\begin{aligned} v_{i \rightarrow j} \simeq \frac{k_i - 1}{\sum _l k_l(k_l-1)}. \end{aligned}$$

In Supplementary Fig. SF5 we check the dependence obtained for the components $v_{i \rightarrow j}$ of the PEV of the NB matrix as a function of the outgoing $k_i$ and ingoing $k_j$ degree, namely $v_{i \rightarrow j} \sim k_i-1$. The averaged components $v_{{\mathrm {out}}}$ and $v_{\mathrm {in}}$, defined in Eqs. (21) and (26), correctly fulfill the scaling forms $v_{{\mathrm {out}}} \sim k -1$ and $v_{{\mathrm {in}}} \sim {\mathrm {const.}}$, respectively. Indeed, for UCM networks, the theoretical predictions in Eqs. (25) and  (28) are extremely well fulfilled.

### Localization of the non-backtracking centrality

The concept of vector localization/delocalization refers to whether the components $x_i$ of a vector are evenly distributed over the network or they attain a large value on some subset of nodes *V* of size $N_V$ and are much smaller in the rest of the network. In the first scenario we have $x_i \sim {\mathrm {const.}}$ for all nodes *i*, and we say the vector is delocalized. In the second scenario, one has $x_i \sim {\mathrm {const.}}$ for $i \in V$, and $x_i \sim 0$ for $i \notin V$, and we say the vector is localized on *V*. For the NBC $x_i$, defined with a Euclidean normalization $\sum _i x_i^2 = 1$, localization can be measured in terms of the inverse participation ratio $Y_4$^2,15^, defined as

30 $$\begin{aligned} Y_4(N) = \sum _i x_i^4. \end{aligned}$$

For a delocalized vector, $x_i \sim N^{-1/2}$, so one has $Y_4(N) \sim N^{-1}$; on the other hand, for a vector localized on a subgraph of size $N_V$, we have $Y_4(N) \sim N_V^{-1}$. Therefore, fitting the inverse participation ratio to a power-law form $Y_4(N) \sim N^{-\alpha }$, a value $\alpha \simeq 1$ indicates delocalization, while $\alpha < 1$ implies localization on a subextensive set of nodes of size $N_V \sim N^\alpha$^31^. In the extreme case of localization on a finite set of nodes (independent of *N*), one has instead $Y_4(N) \sim {\mathrm {const.}}$

The functional form derived for $x_i$ in Eq. (7) helps to explain the localization properties of the NBC for UCM networks observed in Ref.^31^. In Supplementary Fig. SF6 we show a comparison of the inverse participation ratio $Y_4(N)$ numerically obtained in power-law UCM networks with the theoretical prediction computed from Eq. (7), $Y_4^{\mathrm{un}}(N)$, and with the prediction obtained from the annealed network approximation Eq. (4), $Y_4^{\mathrm{an}}(N)$. As we can see, the prediction from our expression, $Y_4^{\mathrm{un}}(N)$, provides an almost perfect match for the numerical observation, while the annealed network approximation exhibits sizeable inaccuracies, particularly in the range $2.5< \gamma < 3.5$.

### Largest non-backtracking eigenvalue of characteristic subgraphs

#### Dangling star graph

Let us consider a dangling star network, see Supplementary Fig. SF1a, formed by a hub *h* of degree *K* connected to $K-1$ leaves *l* of degree 1 and by one edge to a connector node *n* of a generic network. By applying Eq. (15), we obtain the following equations for the LEV $\mu _M$ and the NBC:

31 $$\begin{aligned}&\mu _M [ (K-1)x_l + x_n ] - (K-1)x_h = \mu _M^2 x_h, \end{aligned}$$

32 $$\begin{aligned}&\mu _M x_h = \mu _M^2 x_l,\end{aligned}$$

33 $$\begin{aligned}&\mu _M \left[\sum _{i \ne h} A_{ni} x_i + x_h\right] - k_n x_n = \mu _M^2 x_n, \end{aligned}$$

where $k_n$ is the degree of node *n*, $x_l$ is the NBC centrality of each leaf, and the equations corresponding to the rest of the nodes $i \ne n$ are the same as in the absence of the dangling star.

From the first two equations, assuming $\mu _M \ne 0$, we obtain $x_h = \mu _M x_l$ and $x_n = \mu _M x_h$. Introducing the last equality into the third equation, the dependence on $x_h$ drops out and the equation takes the form of Eq. (15) in the absence of the dangling star. We conclude therefore that a dangling star is unable to alter the value of the overall LEV $\mu _M$ and its NBC depends only on the centrality of the connector node *n*. The reason for this is the absence of non-backtracking paths between the hub and the leaves, so that the hub has the effect of a node of degree one^2,9^.

#### Integrated star graph

The case of an integrated star of degree *K*, i.e., a star connected by *K* edges to *K* randomly chosen connector nodes in a network, Supplementary Fig. SF1b, is more difficult to analyze. To simplify calculations, we consider the case of a regular network with fixed degree *q*. For symmetry reasons, the nodes connected to the hub, of degree $q+1$, have approximately the same NBC, $x_1$, different from the centrality $x_2$ of the nodes not connected to the hub, and also from $x_0$, the centrality of the hub. Applying the Ihara–Bass determinant formula, Eq. (15), we can write

$$\begin{aligned}\mu _M K x_1 &= (K + \mu _M^2 - 1) x_0,\\&\quad\mu _M \left[ x_0 + q\frac{K}{N} x_1 + q\left( 1-\frac{K}{N} \right) x_2 \right] = (q + \mu _M^2) x_1,\\&\quad\mu _M \left[ q\frac{K}{N} x_1 + q \left( 1-\frac{K}{N} \right) x_2\right] = (q + \mu _M^2 -1) x_2,\\ \end{aligned}$$

where to ease calculations, we have made the mean-field assumption that nodes in the network are neighbors of nodes connected to the hub with probability *K*/*N*, and otherwise with probability $1 - K/N$, which is valid in the limit of large *K* and *N*. These conditions lead to the equation for $\mu _M$

34 $$\begin{aligned} \begin{aligned}{}&\mu _M^{5} + \mu _M^{4} \left( 1 - q\right) + \mu _M^{3} \left( q - 1\right) - \mu _M^{2} \left[ \frac{Kq(K-1)}{N} + (q-1)^2 \right] \\&\quad + \mu _M \frac{q(K-1)(N-K)}{N} - q(K-1)(q-1) = 0 \end{aligned} , \end{aligned}$$

where we have factorized the trivial solution $\mu _M = 1$. This is an algebraic equation of fifth order than cannot be solved analytically in general. However, for $K(K-1)q \gg N$, assuming $\mu _M \gg q-1$, it reduces to

35 $$\begin{aligned} \mu _M^5 + \mu _M^2 \frac{Kq(K-1)}{N}= 0, \end{aligned}$$

leading to the solution

36 $$\begin{aligned} \mu _M^{\mathrm {h}} \simeq \left( \frac{q K(K-1)}{N} \right) ^{1/3}. \end{aligned}$$

Instead for $K(K-1)q \ll N$, assuming $\mu _M = q-1+\epsilon$ and expanding Eq. (34) to first order in $\epsilon$, we obtain

37 $$\begin{aligned} \epsilon = \frac{(q-1)^2+(q-1)}{(q-1)^4+(q-1)^3+q(K-1)} \frac{Kq(K-1)}{N}. \end{aligned}$$

Hence the value of $\mu _M$ is very close to the value $q-1$ of the original random regular network, with a correction that vanishes with *N*. We conclude that the addition of a finite integrated hub does not change the value $\mu _M$ of the whole network unless $K(K-1)q \gg N$, a case which may be relevant in small networks. Not surprisingly, the uncorrelated expression Eq. (8) fails here, since it predicts a finite value $\mu _M^{\mathrm{un}} \sim 2 q$, in the limit of large *K*.

While we considered a star integrated into a homogeneous network, Supplementary Fig. SF7 shows that the same picture is valid also in the case of power-law distributed synthetic networks, replacing *q* by the network average degree $\left\langle {k} \right\rangle$: for *K* up to values of the order of $(N/\left\langle {k} \right\rangle )^{1/2}$ the addition of the hub has no effect on $\mu _M$; for larger values, Eq. (36) holds.

#### Overlapping hubs

Let us consider now a graph composed of *n* hubs, sharing all their *K* leaves, see Supplementary Fig. SF1c. We can evaluate $\mu _M$ and $x_i$ by applying again the Ihara–Bass determinant formula. For symmetry reasons, the components $x_h$ of the hubs are equal, and correspondingly the components $x_\ell$ of the leaves. Thus, from Eq. (15) we can write

38 $$\begin{aligned} \mu _M K x_\ell&= (K + \mu _M^2 - 1) x_h, \end{aligned}$$

39 $$\begin{aligned} \mu _M n x_h&= (n + \mu _M^2 - 1) x_\ell , \end{aligned}$$

Imposing that the components $x_h$ and $x_\ell$ are non-zero, we obtain the largest eigenvalue

40 $$\begin{aligned} \mu _M^{\mathrm {oh}} = \sqrt{(n-1)(K-1)}, \end{aligned}$$

while the NB centralities fulfill

41 $$\begin{aligned} \frac{x_\ell ^2}{x_h^2} = \frac{K-1}{K^2} \frac{n^2}{n-1}. \end{aligned}$$

That is, for large *K*, the NBC becomes strongly localized in the hubs.

In Supplementary Fig. SF8 we check the effects of adding *n* overlapping hubs of degree $K$ to power-law distributed synthetic networks. As we can see, as soon as $\mu _M^{\mathrm {oh}}$ is large enough (in practice, when $K > 1 + \left( \frac{\left\langle {k^2} \right\rangle }{\left\langle {k} \right\rangle } -1 \right) ^2/(n-1)$), the actual value of the NB LEV is dominated by the presence of the overlapping hubs.

### *K*-core decomposition

The *K*-core decomposition^32^ is an iterative classification process of the vertices of a network in layers of increasing density of mutual connections, denoted by increasing values of the index *K*. One starts removing the vertices of degree $k=1$, repeating the process until only nodes with degree $k \ge 2$ are left. The removed nodes constitute the $K = 1$ shell, and the remaining ones are the $K = 2$ core. At the next step, all vertices with degree $k=2$ are iteratively removed, thus leaving the $K = 3$ core. The procedure is repeated until the maximum *K*-core (of index $K_M$) is reached, such that one more iteration removes all nodes in the network. The maximum *K*-core of generic networks is usually a homogeneous subgraph^23^. The *K*-core structure of networks has been proposed as a classification of node importance in dynamical processes on complex topologies^33^.

### Algorithm to determine optimal *n* and *K* values for overlapping hubs

The determination of the set of all overlapping hubs in a real-world network is highly time consuming. We can however obtain a working approximation using the following greedy algorithm: We order the nodes in decreasing order of their degree, $i_1, i_2, \ldots , i_N$. Starting from node $i_\alpha$, we visit the set of nodes $i_\alpha , i_{\alpha +1}, \ldots i_{\alpha +q}$ and identify and identify the number of common neighbors $K_q^\alpha$, that are common neighbors of the set of nodes $i_\alpha , i_{\alpha +1}, \ldots i_{\alpha +q}$. Repeating this process for all nodes in the network, we compute the values $K_q^\alpha$ for all nodes $\alpha$ and all sets of nodes (in decreasing order of degree) of length $q +1$. We choose as values of *n* and *K* the values of $q+1$ and $K_q^\alpha$ that maximize the product $q (K_q^\alpha -1)$.

### Numerical simulations of bond percolation

We consider the bond percolation process in which network edges are randomly kept with probability *p* and removed with probability $1-p$. For each realization of this process with a given value of *p*, one considers the largest cluster remaining in the network, of size $S_p$. The average of this quantity over independent realization is denoted by $\left\langle {S_p} \right\rangle$. The critical percolation point $p_c$ separates a subcritical phase at $p < p_c$, in which only clusters of small size are present, so that $\left\langle {S_p} \right\rangle / N \rightarrow 0$ in the thermodynamic limit $N\rightarrow \infty$, from a supercritical phase at $p> p_c$, in which there is a finite spanning cluster leading to $\left\langle {S_p} \right\rangle /N \rightarrow {\mathrm {const.}}$^34^.

In order to estimate the value of the percolation point, one considers the susceptibility $\chi _2(p)$, defined as^18,35^

42 $$\begin{aligned} \chi _2(p) = \frac{\left\langle {S_p^2} \right\rangle - \left\langle {S_p} \right\rangle ^2}{\left\langle {S_p} \right\rangle }. \end{aligned}$$

The percolation threshold $p_c$ is defined as the value of *p* for which $\chi _2(p)$ shows a maximum^35^. To compute numerically $\chi _2(p)$ in real-world networks we perform the averages on bond percolation experiments applying the Newman-Ziff algorithm^36^.

## Supplementary information

Supplementary Information 1
