# Supplementary material for: The localization of non-backtracking centrality in networks and its physical consequences
Source: Sci Rep. 2020 Dec 10;10:21639. doi: 10.1038/s41598-020-78582-x (PMC7728761; doi:10.1038/s41598-020-78582-x)
Supplement: Supplementary file 1 — Supplementary Information 1 [file 41598_2020_78582_MOESM1_ESM.pdf]

SUPPLEMENTARY INFORMATION FOR
The localization of non-backtracking centrality in networks and its physical consequences

Romualdo Pastor-Satorras[1] and Claudio Castellano[2]

[1]*Departament de Física, Universitat Politècnica de Catalunya,*
Campus Nord B4, 08034 Barcelona, Spain

[2]*Istituto dei Sistemi Complessi (ISC-CNR),*
Via dei Taurini 19, I-00185 Roma, Italy

Corresponding author: romualdo.pastor@upc.edu

SUPPLEMENTARY TABLE

Supplementary Table ST1: Topological and spectral properties of the 109 real-world networks in Ref. [18] for which we test our theory. We report the following properties of this set of networks: N: network size; $\langle k \rangle$: average degree; $k_{\max}$: maximum degree; μ_M: LEV of the NBC; μ_M^{an}: theoretical approximation for μ_M within the annealed network approximation, Eq. (9); μ_M^{un}: theoretical approximation for μ_M in uncorrelated networks, Eq. (8); μ_M^{oh}: theoretical approximation for μ_M taking into account the effect of overlapping hubs, Eq. (10); μ_M^{core}: LEV of the NBC for the maximum K-core of the network; p_c^{-1}: inverse of the numerical percolation threshold p_c, estimated as the position of the principal peak of the susceptibility χ_2.

	Network	N	$\langle k \rangle$	$k_{\max}$	μ_M	μ_M^{an}	μ_M^{un}	μ_M^{oh}	μ_M^{core}	p_c^{-1}
0	Social 3	32	5.00	13	4.74	4.94	4.76	1.41	3.96	4.0572
1	Karate club	34	4.59	17	5.29	6.77	4.75	2.00	4.18	4.3472
2	Protein 2	53	4.64	8	4.68	4.39	4.52	1.73	4.29	2.9545
3	Dolphins	62	5.13	12	5.99	5.81	5.75	2.00	5.74	4.1694
4	Social 1	67	4.24	11	4.36	4.25	4.38	1.73	3.00	3.2801
5	Les Miserables	77	6.60	36	10.75	11.06	10.04	5.29	9.40	7.5977
6	Protein 1	95	4.48	7	4.25	3.95	4.01	1.41	4.23	1.7169
7	E. Coli, transcription	97	4.37	10	5.34	4.41	4.86	1.73	4.83	1.9855
8	Political books	105	8.40	25	10.63	10.93	10.40	3.61	8.97	5.4306
9	David Copperfield	112	7.59	49	11.54	12.77	11.44	4.47	10.32	9.2696
10	College football	115	10.66	12	9.77	9.73	9.75	3.46	9.75	7.3987
11	S 208	122	3.10	10	2.75	2.77	2.76	1.00	2.75	2.1438
12	High school, 2011	126	27.13	55	32.85	31.79	32.13	9.59	26.09	25.8514
13	Bay Dry	128	32.42	110	38.44	39.11	38.04	13.86	34.69	31.7995

	Network	N	$\langle k \rangle$	$k_{\max}$	μ_M	$\mu_M^{\rm an}$	$\mu_M^{\rm un}$	$\mu_M^{\rm oh}$	$\mu_M^{\rm core}$	p_c^{-1}
14	Bay Wet	128	32.91	110	38.91	39.50	38.53	12.37	33.64	32.3371
15	Radoslaw Email	167	38.92	139	59.43	63.46	58.35	26.72	52.81	49.4981
16	High school, 2012	180	24.67	56	29.01	28.55	28.72	6.93	22.69	22.5561
17	Little Rock Lake	183	26.60	105	40.06	41.89	38.37	16.12	34.70	32.4229
18	Jazz	198	27.70	100	38.82	37.64	37.83	10.39	28.00	30.6470
19	S 420	252	3.17	14	2.89	2.91	2.90	1.00	2.89	2.2160
20	C. Elegans, neural	297	14.46	134	22.76	25.05	20.87	7.35	20.02	18.1451
21	Network Science	379	4.82	34	8.71	7.02	6.53	4.00	7.00	2.5558
22	Dublin	410	13.49	50	22.24	17.72	18.75	6.00	21.31	12.9029
23	US Air Trasportation	500	11.92	145	46.54	52.78	43.04	15.30	31.58	37.8733
24	S 838	512	3.20	22	2.94	3.03	2.96	1.00	2.94	2.2063
25	Yeast, transcription	662	3.21	71	6.50	12.51	3.02	5.57	5.09	4.1265
26	URV email	1133	9.62	71	19.27	17.69	18.37	4.00	10.00	15.4127
27	Political blogs	1222	27.36	351	72.56	80.26	66.72	13.82	42.71	58.9779
28	Air traffic	1226	3.93	34	7.48	6.36	6.26	2.24	6.70	5.4898
29	Yeast, protein	1458	2.67	56	5.05	6.13	3.25	1.00	4.00	3.3193
30	Petster, hamster	1788	13.96	272	44.31	44.55	40.19	14.00	34.73	36.3558
31	UC Irvine	1893	14.62	255	46.25	54.64	43.70	9.27	35.39	39.2625
32	Yeast, protein	2172	6.05	215	18.54	18.79	16.31	4.36	10.66	13.6990
33	Japanese	2698	5.93	725	38.16	107.61	23.46	14.32	23.56	30.7833
34	Open flights	2905	10.77	242	61.33	54.84	57.28	14.14	32.02	49.2010
35	GR-QC, 1993-2003	4158	6.46	81	44.44	16.98	27.64	20.20	42.00	7.4308

	Network	N	$\langle k\rangle$	$k_{\max}$	μ_M	$\mu_M^{\rm an}$	$\mu_M^{\rm un}$	$\mu_M^{\rm oh}$	$\mu_M^{\rm core}$	p_c^{-1}
36	Tennis	4338	37.74	451	160.17	157.91	158.09	17.38	124.14	136.0226
37	US Power grid	4941	2.67	19	6.23	2.87	2.88	1.41	5.06	1.5142
38	HT09	5352	6.91	1287	41.01	198.98	9.06	13.27	25.42	34.8533
39	Hep-Th, 1995-1999	5835	4.74	50	17.01	8.12	9.41	6.48	17.00	9.0419
40	Reactome	5973	48.81	855	206.88	142.31	160.58	91.04	197.41	87.9832
41	Jung	6120	16.43	5655	128.35	990.77	29.33	103.36	77.46	107.0054
42	Gnutella, Aug. 8, 2002	6299	6.60	97	26.51	16.66	17.60	4.69	22.35	22.0829
43	JDK	6434	16.68	5923	129.28	981.71	29.92	103.74	77.46	107.1269
44	AS Oregon	6474	3.88	1458	35.04	163.81	14.68	18.52	14.96	28.0308
45	English	7377	11.98	2568	104.34	319.70	59.17	32.83	58.34	87.9832
46	Gnutella, Aug. 9, 2002	8104	6.42	102	26.56	15.82	16.65	5.10	23.39	21.9957
47	French	8308	5.74	1891	52.46	217.01	26.58	18.49	23.12	43.1321
48	Hep-Th, 1993-2003	8638	5.74	65	30.01	11.99	14.42	13.75	30.00	13.2956
49	Gnutella, Aug. 6, 2002	8717	7.23	115	20.47	13.40	14.02	8.37	16.94	15.0067
50	Gnutella, Aug. 5, 2002	8842	7.20	88	21.58	13.79	14.01	5.29	18.62	17.2084
51	PGP	10680	4.55	205	41.03	17.88	26.19	9.49	35.73	14.6018
52	Gnutella, August 4 2002	10876	7.35	103	15.28	12.97	12.86	4.36	13.19	12.9654
53	Hep-Ph, 1993-2003	11204	21.00	491	243.75	129.88	206.61	113.67	237.00	209.4101
54	Spanish	11558	7.45	2986	93.51	456.58	40.68	32.19	44.11	78.1537
55	DBLP, citations	12495	7.93	709	38.06	42.77	33.58	14.56	31.19	30.2164
56	Spanish	12643	8.70	5169	100.13	806.66	28.19	35.37	47.63	83.9067
57	Cond-Mat, 1995-1999	13861	6.44	107	23.14	12.54	14.83	6.00	16.00	15.5067

	Network	N	$\langle k \rangle$	$k_{\max}$	μ_M	μ_M^{an}	μ_M^{un}	μ_M^{oh}	μ_M^{core}	p_c^{-1}
58	Astrophysics	14845	16.12	360	72.21	44.46	55.60	19.60	55.00	55.0700
59	Google	15763	18.85	11401	156.61	900.63	47.71	86.99	106.57	125.5953
60	AstroPhys, 1993-2003	17903	22.00	504	92.54	64.70	77.74	16.55	55.00	76.5451
61	Cond-Mat, 1993-2003	21363	8.55	279	35.80	21.47	26.02	12.73	24.00	27.3670
62	Gnutella, Aug. 25, 2002	22663	4.83	66	9.38	9.75	8.96	2.45	8.87	8.6900
63	Internet	22963	4.22	2390	64.68	260.46	28.28	24.25	39.97	51.4491
64	Thesaurus	23132	25.69	1062	97.70	102.29	94.53	15.17	82.91	88.5512
65	Cora	23166	7.70	377	29.28	22.68	19.42	8.06	16.91	21.9551
66	Linux, mailing list	24567	12.88	2989	220.15	339.98	178.45	46.66	121.12	190.7713
67	AS Caida	26475	4.03	2628	59.41	279.24	26.29	24.62	34.41	48.1394
68	Gnutella, Aug. 24, 2002	26498	4.93	355	10.78	11.03	10.77	2.24	10.34	9.4122
69	Hep-Th, citations	27400	25.69	2468	106.82	105.40	88.46	54.94	43.36	90.5805
70	Cond-Mat, 1995-2003	27519	8.44	202	38.30	21.29	26.52	12.45	23.00	29.3631
71	Digg	29652	5.72	283	27.63	27.07	27.22	4.24	23.52	24.1307
72	Linux, soft.	30817	13.84	9338	154.98	851.62	34.55	69.53	58.94	129.6788
73	Enron	33696	10.73	1383	115.48	141.36	90.59	15.30	79.03	99.2556
74	Hep-Ph, citations	34401	24.46	846	74.33	62.50	61.91	19.85	33.57	63.3955
75	Cond-Mat, 1995-2005	36458	9.42	278	49.17	26.88	34.58	14.66	28.00	38.8247
76	Gnutella, Aug. 30, 2002	36646	4.82	55	11.39	10.46	9.93	2.83	6.00	10.2897
77	Slashdot	51083	4.56	2915	44.95	80.57	34.72	9.49	35.63	37.7581
78	Gnutella, Aug. 31, 2002	62561	4.73	95	11.48	10.60	10.05	2.00	9.57	10.4354
79	Facebook	63392	25.77	1098	130.82	87.05	105.41	12.37	100.56	114.5491

	Network	N	$\langle k \rangle$	$k_{\max}$	μ_M	μ_M^{an}	μ_M^{un}	μ_M^{oh}	μ_M^{core}	p_c^{-1}
80	Epinions	75877	10.69	3044	181.65	182.88	161.62	23.22	129.54	161.1385
81	Slashdot zoo	79116	11.82	2534	127.57	145.30	106.05	23.94	80.90	112.1438
82	Flickr	105722	43.83	5425	614.42	348.21	429.50	68.08	572.00	71.8799
83	Wikipedia, edits	113123	35.82	20153	389.69	688.54	289.34	96.31	216.89	347.9713
84	Petster, cats	148826	73.21	80634	1160.43	9291.62	261.40	873.92	664.31	1017.3354
85	Gowalla	196591	9.67	14730	159.86	305.58	76.47	35.33	81.48	136.8895
86	Libimseti	220970	155.98	33389	943.38	1639.96	671.28	140.18	572.24	882.0520
87	EU email	224832	3.02	7636	97.09	566.65	26.93	9.85	72.95	82.2030
88	Web Stanford	255265	15.21	38625	423.82	2029.74	46.88	336.93	130.43	18.0571
89	Amazon, Mar. 2, 2003	262111	6.87	420	17.80	10.14	10.04	5.10	7.92	10.5122
90	DBLP, collaborations	317080	6.62	343	114.72	20.75	31.33	40.00	112.00	29.5135
91	Web Notre Dame	325729	6.69	10721	175.66	279.68	55.53	70.99	164.69	12.3073
92	MathSciNet	332689	4.93	496	33.53	15.43	18.85	6.71	23.00	21.0558
93	CiteSeer	365154	9.43	1739	52.54	47.45	29.49	10.25	35.33	40.4606
94	Zhishi	372840	12.43	127066	942.98	27908.59	15.43	942.62	295.29	33.1929
95	Actor coll. net.	374511	80.18	3956	847.55	417.32	573.14	61.48	592.15	776.8318
96	Amazon, Mar. 12, 2003	400727	11.73	2747	35.03	29.33	20.30	16.25	31.68	24.9309
97	Amazon, Jun. 6, 2003	403364	12.11	2752	40.31	29.55	21.73	17.15	33.06	27.5921
98	Amazon, May 5, 2003	410236	11.89	2760	40.36	29.93	21.81	17.38	32.59	27.6319
99	Petster, dogs	426485	40.06	46503	734.01	2054.76	363.83	427.47	427.52	665.8499
100	Road network PA	1087562	2.83	9	3.11	2.20	2.24	1.41	2.90	1.4442
101	YouTube friend. net.	1134890	5.27	28754	185.14	493.53	80.41	56.99	105.78	156.6161

Network	N	$\langle k \rangle$	$k_{\max}$	μ_M	μ_M^{an}	μ_M^{un}	μ_M^{oh}	μ_M^{core}	p_c^{-1}
102 Road network TX	1351137	2.78	12	3.56	2.15	2.19	1.41	3.51	1.3623
103 AS Skitter	1694616	13.09	35455	653.66	1444.15	89.41	260.77	154.76	563.5708
104 Road network CA	1957027	2.82	12	3.32	2.17	2.21	1.41	3.17	1.4409
105 Wikipedia, pages	2070367	40.90	230040	775.44	3345.71	308.90	190.21	302.35	699.8880
106 US Patents	3764117	8.77	793	110.45	20.34	27.28	55.41	75.82	34.3938
107 DBpedia	3915921	6.42	469692	462.92	13856.37	17.53	388.33	28.30	58.5652
108 LiveJournal	5189808	18.76	15016	537.93	154.42	221.84	43.89	408.16	361.7945

SUPPLEMENTARY FIGURES

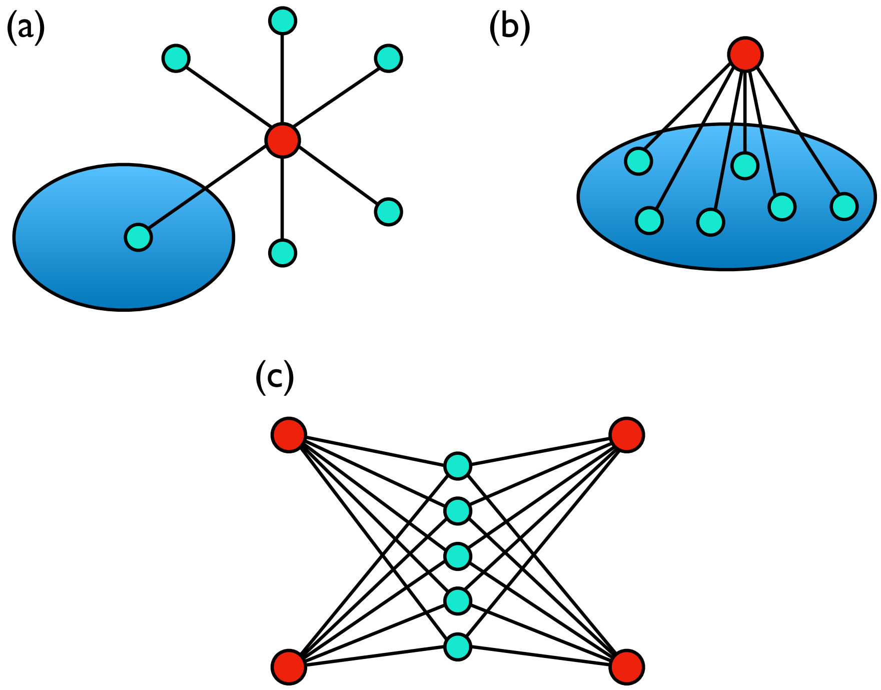

Supplementary Figure SF1. Graphical representation of star subgraphs. (a) Dangling hub of degree K, connected to $K-1$ leaves of degree 1 and to a connector node in a generic network. $K = 6$. (b) Integrated hub of degree K connected to K connector nodes in a generic network. $K = 6$. (c) Example of n overlapping hubs of degree K, sharing the same set of leaves of degree n. $n = 4$, $K = 5$.

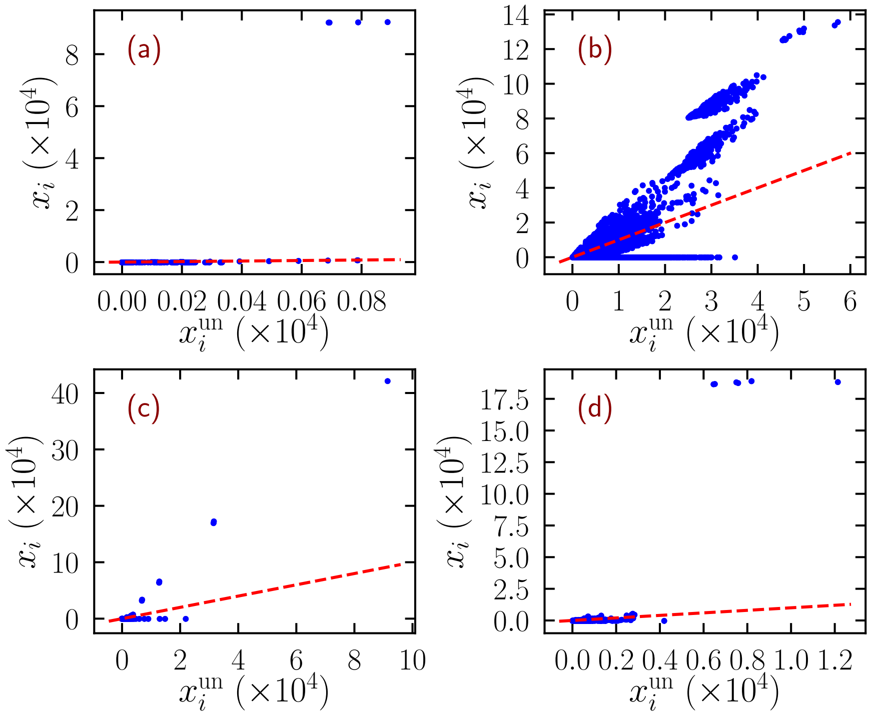

Supplementary Figure SF2. **NBC localization in real-world networks.** Scatter plot of the NBC x_i as a function of the theoretical prediction x_i^{un}, Eq. (7) in four examples of real-world networks (a) `Zhishi`; (b) `Flickr`; (c) `Web Notre Dame`; (d) `Web Stanford`. The dashed line represents the behavior $y = x$.

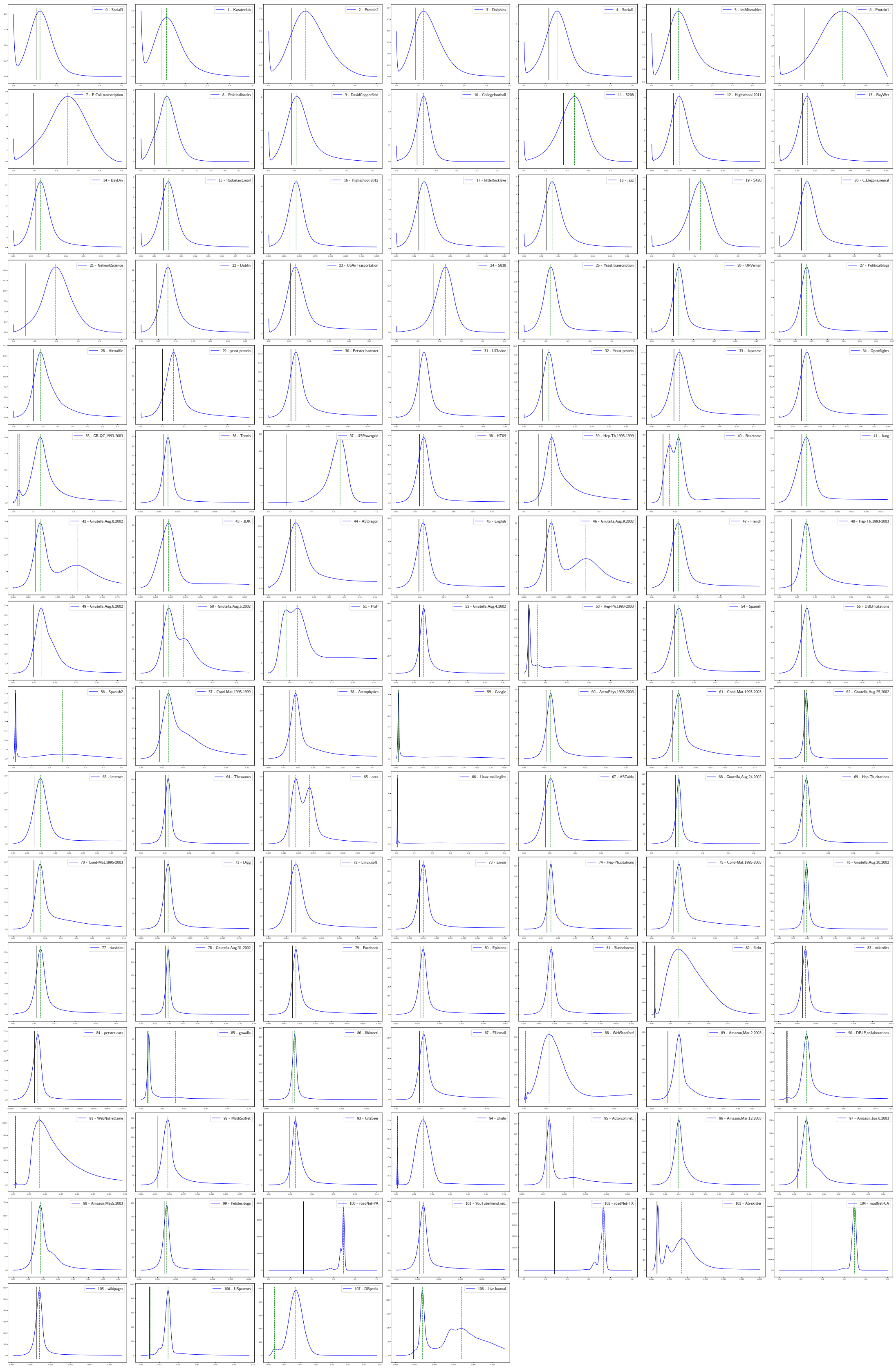

Supplementary Figure SF3. **Susceptibility $\chi_2(p)$ for all 109 networks considered.** In each plot, the green dashed vertical line(s) denote the position(s) of the peak(s), the black continuous vertical line denotes the value of $1/\mu_M$.

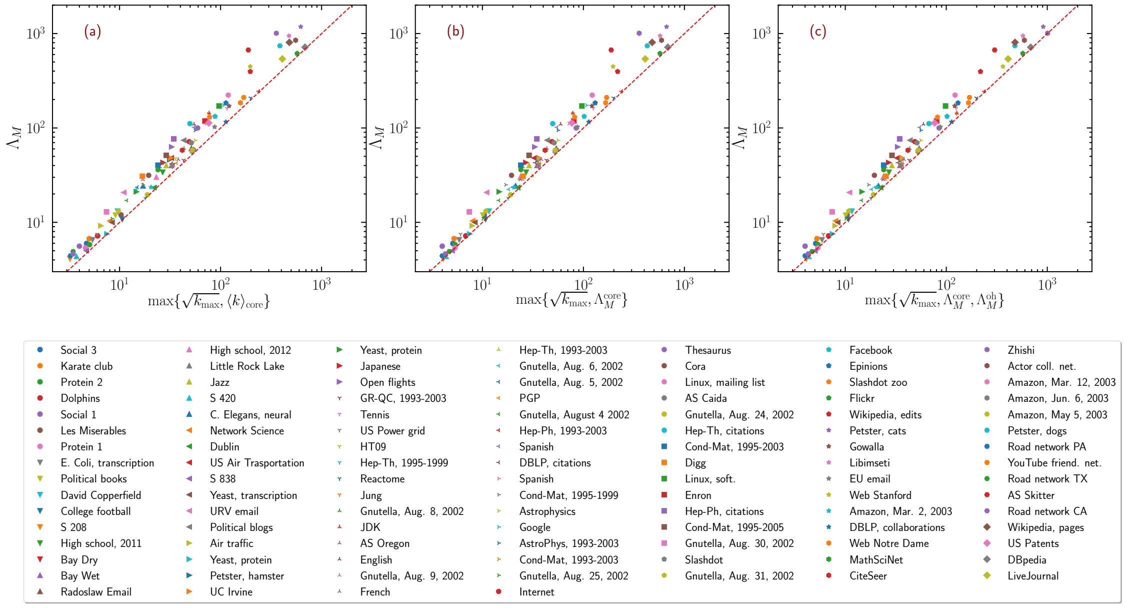

Supplementary Figure SF4. **Check of the theoretical approximations for the LEV of the adjacency matrix in real networks.** (a) Value of the LEV Λ_M of the adjacency matrix as a function of the theoretical prediction in Ref. [23], given as the maximum between the square root of the maximum degree and the LEV $\Lambda_M^{\rm core}$of the maximum K-core, approximated by its average degree $\langle k \rangle_{\rm core}$; (b) same expression, considering the LEV of the maximum K-core computed numerically; (c) an improved version taking into account the LEV $\Lambda_M^{\rm oh}$ of the maximal set of n overlapping hubs of degree K.

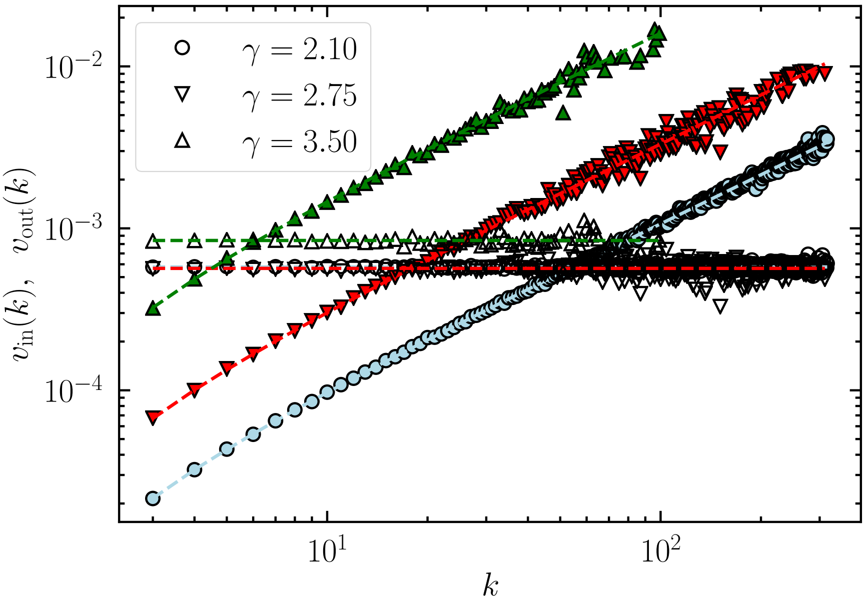

Supplementary Figure SF5. **Behavior of $v_{\rm out}(k)$ and $v_{\rm in}(k)$.** Check of the scaling of $v_{\rm out}(k)$ (filled symbols) and $v_{\rm in}(k)$ (hollow symbols) with degree k in power-law UCM networks of size $N = 10^5$ and different γ exponents. Dashed lines denote the theoretical behaviors predicted for $v_{\rm out}(k)$, Eq. (25) and for $v_{\rm in}(k)$, Eq. (28).

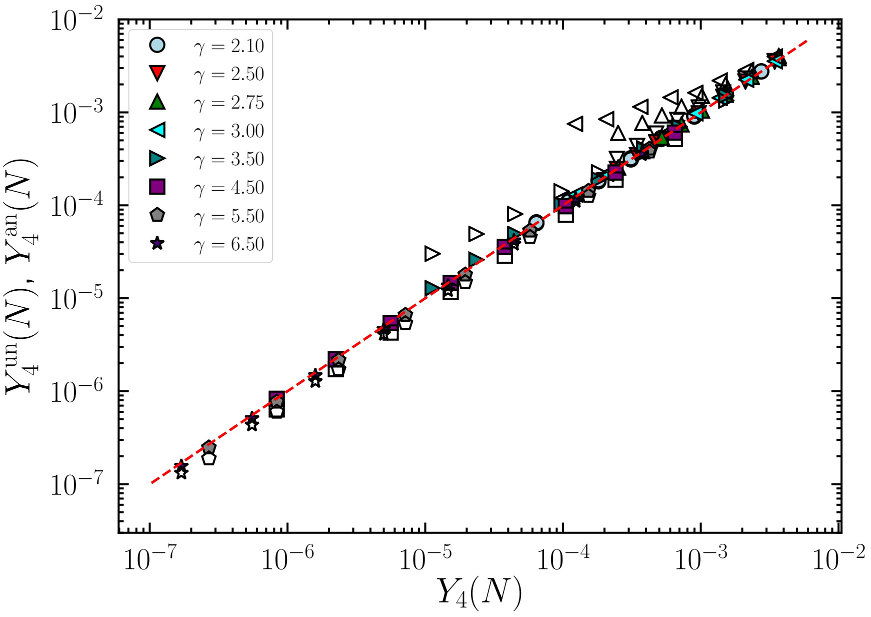

Supplementary Figure SF6. **Localization in synthetic uncorrelated networks.** Inverse participation ratio $Y_4(N)$ of the NBC x_i in power-law UCM networks with different degree exponent γ. We compare, for different network sizes, the results from numerical evaluation with the theoretical prediction $Y_4^{\mathrm{un}}(N)$ computed from the expression $x_i \sim \sum_j A_{ij}(k_j - 1)$ (full symbols), and with the prediction $Y_4^{\mathrm{an}}(N)$ from the annealed network approximation $x_i \sim k_i$ (hollow symbols). The dashed line represents the behavior $y = x$. Simulations results correspond to the average over 25 different network realizations of sizes ranging between $N = 3000$ and $N = 10^7$.

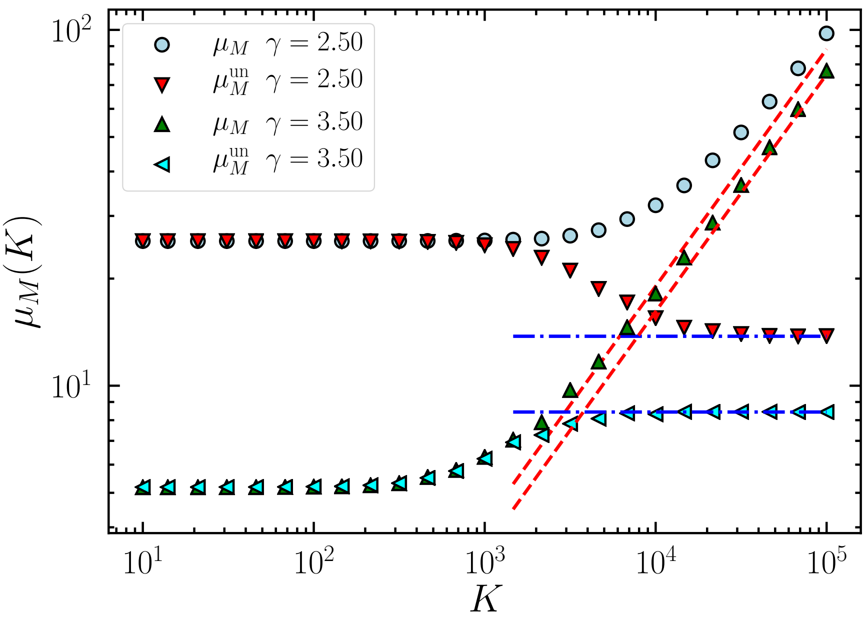

Supplementary Figure SF7. Effects of the addition of an integrated hub. Value of μ_M for power-law UCM networks with different degree exponent added with an integreated hub of degree K. Dashed lines represent the theoretical prediction, $\mu_M^{\rm h} = \left(\frac{\langle k \rangle K(K-1)}{N}\right)^{1/3}$. Dot-dashed lines represent the estimation $\mu_M^{\rm un} \sim 2\langle k \rangle$, large values of K according to the uncorrelated theory, Eq. (8). Network size $N = 10^5$.

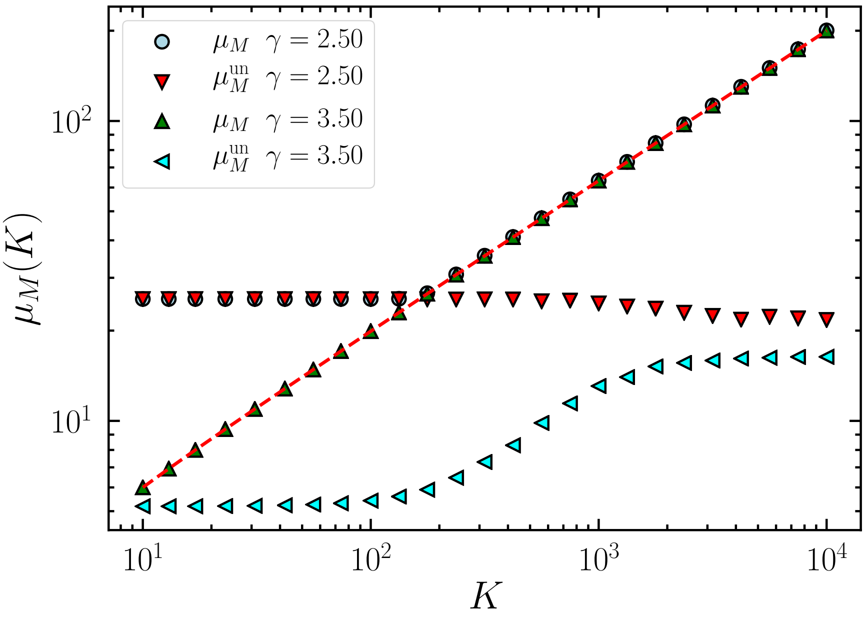

Supplementary Figure SF8. **Effect of the addition of overlapping hubs.** Value of μ_M for power-law UCM networks with different degree exponent, added with $n = 5$ overlapping hubs of degree K. The dashed line represents the theoretical prediction, $\mu_M^{\rm oh} = [(n-1)(K-1)]^{1/2}$, independent of γ. Network size $N = 10^5$.
